# Stress Distribution on Various Implant-Retained Bar Overdentures

**DOI:** 10.3390/ma15093248

**Published:** 2022-04-30

**Authors:** Övül Kümbüloğlu, Beril Koyuncu, Gözde Yerlioğlu, Nadin Al-Haj Husain, Mutlu Özcan

**Affiliations:** 1 Department of Prosthodontics, Ege University Faculty of Dentistry, Izmir 35040, Turkey; t_ovul@yahoo.com (Ö.K.); gzdyerlioglu@gmail.com (G.Y.); 2 Private Practice at RadixDent, Hurriyet Road, Kordonboyu, No:60, Kartal 34860, Turkey; 3 Department of Reconstructive Dentistry and Gerodontology, School of Dental Medicine, University of Bern, 3010 Bern, Switzerland; nadin.al-haj-husain@zmk.unibe.ch; 4 Division of Dental Biomaterials, Center of Dental Medicine, Clinic for Reconstructive Dentistry, University of Zurich, 8032 Zurich, Switzerland; mutluozcan@hotmail.com

**Keywords:** finite element analysis, CNC milling, hader bar, implant-supported mandibular overdenture

## Abstract

The purpose of this study was to evaluate the effects of various fabrication techniques and materials used in implant-supported mandibular overdentures with a Hader bar attachment over added stress distribution. Three-dimensional geometric solid models, consisting of two implants (3.3 mm × 12 mm) placed at the bone level on both mandibular canine regions and a Hader bar structure, were prepared. Model 1 simulated a bar retentive system made from Titanium Grade 5 material by Computer Numerical Control (CNC) milling technique without using any converting adapter/multi-unit element on the implants, while Model 2 simulated the same configuration, but with converting adapters on the implants. Model 3 simulated a bar retentive system made from Cobalt-Chromium material, made by using conventional casting technique with converting adapters on the implants. Static loads of 100 Newton were applied on test models from horizontal, vertical and oblique directions. ANSYS R15.0 Workbench Software was used to compare Von Mises stress distribution and minimum/maximum principal stress values, and the results were evaluated by using Finite Element Analysis method. As a result, the highest stress distribution values under static loading in three different directions were obtained in Model 1. Stress was observed intensely around the necks of the implants and the surrounding cortical bone areas in all models. In scope of the results obtained, using converting adapters on implants has been considered to decrease transmission of forces onto implants and surrounding bone structures, thus providing a better stress distribution. It has also been observed that the type of material used for bar fabrication has no significant influence on stress values in those models where converting adapters were used.

## 1. Introduction

Mandibular complete dentures are still preferred for the treatment of mandibular edentulous patients although the patient satisfaction is known to be quite low. Currently, implant therapy is quite popular for different choices according to patient characteristics and demands. This therapy also overcomes the retention and comfort problems of a conventional mandibular complete denture, improving the patient’s quality of life [1]. There are two possible types of implant therapies for edentulous patients: implant-supported restorations or implant-retained overdentures. In some mandibular edentulous cases, due to severe bone resorption in the posterior area, and the location of the inferior alveolar nerve, implant placement may not be possible. Therefore, sometimes a biomechanically correct fixed restoration cannot be planned [2]. The placement of a minimum of two implants in the interforaminal region, where bone resorption is not usually severe, and an implant-retained overdenture may increase the retention and stability of prosthesis, mastication performance and patient satisfaction [2,3,4]. In accordance with the 2002 McGill consensus, an overdenture retained with two implants is the preliminary therapy recommended for an edentulous mandible [5]. Bar-clip and stud precision attachments are the two main groups of retention systems that are commonly preferred in implant overdentures. Ball attachments, locators and magnets are examples of the most-preferred individual attachments [1,6]. To increase stability and support under functional loading, splinting of the implants with bar attachments is advised [7].

By using bar-retained overdentures, retention and stability problems that are observed in conventional complete dentures are mainly solved [8,9]. Bar-clip attachments are commonly preferred for advantages such as the distribution of stress on the retaining implants and the bone via denture base plates. In accordance with the remaining bone quality and quantity, two, three or four implants can be used for support. An adequate vertical dimension is a necessity for the use of these attachments [1,10]. Ideal restorative space for bar-clip overdentures should accommodate the denture base, the acrylic teeth and the bar-clip attachment system. A minimum of 13–14 mm space is required between the implant platform and the incisal plane. 4 mm of this space should be arranged for the bar attachment, with a hygiene space of 1 mm under the bar [11]. Another requirement is adequate inter-implant distance, which should be a minimum of 10–12 mm. If the inter-implant distance is less, a milled bar is indicated with frictional fit components, in order to increase the retention [12].

Various metals and alloys are commonly used in the fabrication of bar attachments. In the past, gold alloys were preferred but, due to its expensiveness and flexibility, this material is no longer preferred. Alternative materials for the fabrication of metal bar frameworks are: cobalt-chromium (Co-Cr), titanium (Ti) and silver-palladium (Ag-Pd) alloys [13]. In cases of metal allergy or patients demanding non-metallic restorations, polyetherketoneketone (PEKK) material is a possible low-cost option, although more studies based on the fracture resistance of this novel material must be conducted [14]. Polyetheretherketone (PEEK) belongs to the same polymer family, and can be used as an alternative for a non-metallic framework, with its low-weight advantage [15]. Fibre-reinforced resin (Trinia) is another non-metal framework option with good stress distribution [16].

During the manufacturing of a conventional cast-bar attachment, dimensional changes can occur, associated with the impression-making and fabrication of an accurate dental cast, as well as the lost-wax casting protocol. These dimensional changes (if poorly controlled) risk causing distortion [17,18,19,20,21,22]. Using Computer-Aided Design/Computer-Aided Manufacturing (CAD/CAM) technology in overdenture prosthesis provides a computerised virtual bar design and modification, eliminating the laboratory steps such as casting and modelling. A good, passive fit, lower distortion ratio and long-term success are observed in bar-retained overdentures produced with this technique [23]. However, the use of CAD/CAM technology and CNC Milling in bar overdenture fabrication results in high costs compared to the conventional casting method [24].

Multi-unit abutment is the prosthetic component that facilitates the implant rehabilitation of edentulous or partially edentulous patients. The short height design and the wide margin of the element provide an ease in seating of the framework and restoration. The passive fit of the framework seated on the multi-unit abutments contributes to stress absorbance. These components also have the advantage of solving the inclination problems with angulated choices [25].

This study aimed to compare various fabrication techniques and materials on stress distribution in Hader Bar-retained mandibular overdentures in edentulous mandibles using three-dimensional finite element analysis (3D-FEA). The region of the canines was the preferred location of the implant insertion, in accordance with previous related studies and World Health Organization (WHO) guidelines for totally edentulous patients to be treated with implants. The guidelines advised that a minimum of two implants, inserted in the region of the canines, would be an appropriate treatment plan for these patients [26,27,28,29,30,31,32,33].

## 2. Materials and Methods

In this study, 2 implants were placed symmetrically and parallel to each other at the region of the mandibular canines in an edentulous mandible [34,35,36]. The research was carried out by 3D-FEA to analyse the stress distribution on the implants and bone. An Intel Core i7 3730 3.40 Ghz processor-16 GB RAM-1 TB Hard drive & Windows 7 professional operating system computer and an ANSYS R15.0 Workbench (Swanson Analysis Systems, Inc, Houston, TX, USA) Finite Element Analysis Program were used for the FEA.

A solid model of the interforaminal region of an edentulous mandible was prepared. D2 type of bone is usually observed in the anterior mandible, and for this reason, solid models consisting of 2 mm of cortical bone [37] covering the trabecular part and 1 mm of mucosa covering the cortical bone were prepared. The materials were linearly elastic, isotropic and homogeneous. Two bone-level implants (3.3 mm × 12 mm) from ITI Straumann (Institut Straumann AG, Basel, Switzerland) were used for the modelling. Catia (Kingston, NY, USA) and Solidworks 2014 (D’assault Systems, Waltham, MA, USA) programs were used to create the 3-D mathematical models of the implant, screw, spacer, bar attachment, cortical and spongiosis bone. The implants were inserted in the bone at an angle of 90 degrees, and were assumed to be fully osseointegrated (%100). 20 mm of space was arranged between the implants on the model [38]. The bar attachment was designed as a Hader bar keyhole on the implants [38,39,40]. All of the elasticity modulus and the Poisson ratio of the materials used on these mathematical solid models while performing the FEA were defined on the program. Table 1 shows the elasticity modulus and the Poisson ratio of each material used in this study. The values were obtained from the previous related literature [41,42,43,44,45,46,47].

### 2.1. Preparation of the Solid Models

**Figure 1:**
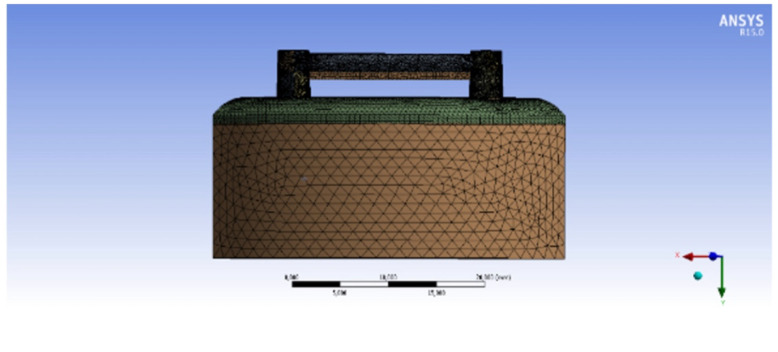
Model 2 after being meshed into elements.

Three different solid models were prepared, consisting of three different bar designs on two symmetrically placed implants in the interforaminal region of an edentulous mandible. Model 1 simulated a bar retentive system made of Ti Grade 5 (Ti-6Al-4V) material by Computer Numerical Control (CNC) milling technique without using any multi-unit abutment on implants, while Model 2 simulated the same configuration, with multi-unit abutments on implants. Model 3 simulated a bar retentive system made of Co-Cr material using conventional casting technique with multi-unit abutments on implants. All the models were meshed into nodes and triangular elements. Model 1 was meshed into 825,425 nodes and 534,756 triangular elements whereas Model 2 had up to 967,773 nodes and 649,221 triangular elements. Model 3 consisted of 1,015,711 nodes and 683,345 triangular elements. An example for the meshed model is shown in Figure 1.

### 2.2. Loading Conditions

**Figure 2:**
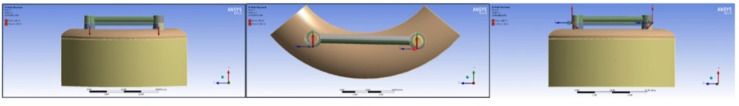
Loads of 100 N in vertical direction, bucco-lingual direction and oblique direction, respectively.

In order to examine the stress distribution around the implants, the bone and the bar attachments, three types of static loads were applied symmetrically on both implants from horizontal, vertical and oblique directions. Static loads of 100 N [46,48,49] were applied to the bar attachment screw in a vertical direction, to the bar attachment abutment in a bucco-lingual direction and on the distal side of the bar attachment abutment with a 45-degree angle in an oblique direction (Figure 2).

## 3. Results

### 3.1. Application of 100 N Static Load

The Von Mises stress is the criteria for the yield strength, whereas the maximum principal stress shows the tensile strength. The tensile strength and the yield strength of the materials used in this study are shown in Table 2 [50]. The Von Mises stress on the implants, the cortical bone and the trabecular bone are shown in Table 3.

### 3.2. Vertical Loading

**Figure 3:**
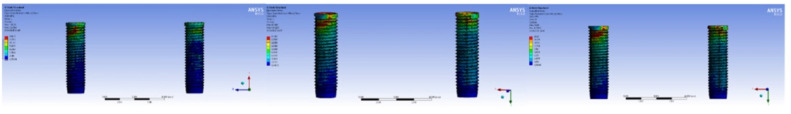
Model 1, Model 2 and Model 3 implants, and the distribution of Von Mises stress after vertical loading, respectively.

The highest Von Mises stress value on the implants was observed in Model 1 (148.24 MPa). The Von Mises stress on the implants was in the range of the Ti yield strength values, and this was the reason why no deformation occurred. Figure 3 exhibits the stress distribution on the implants in Model 1, Model 2 and Model 3. Model 2 (87.49 MPa) and Model 3 (86.86 MPa) did not differ significantly in regard to their Von Mises stress values. This means that the elasticity modulus of the bar attachment materials does not affect the amount and distribution of the stress. Moreover, models that consist of multi-unit abutments have lower stress values because the multi-unit abutment absorbs the forces, providing a decrease in the amount of stress born by the implants.

**Figure 4:**
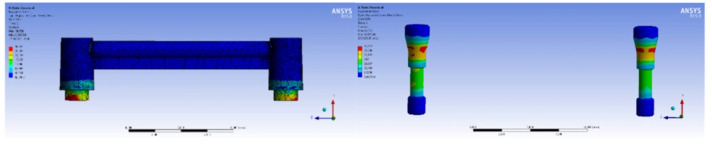
The distribution of Von Mises stress after vertical loading on Model 1 bar attachment and screw.

The highest Von Mises stress on the bar attachment was observed in Model 1 (50.76 MPa), and occurred mostly on the neck part of the bar inside the implant and neck of the screw attaching the bar and implant (62.77 MPa). Figure 4 exhibits the stress distribution on the bar attachment and screw, respectively. The stress on the bar attachment and screw were in the range of physiological limits, and for this reason no damage occurred. The highest stresses on the multi-unit abutments of Model 2 and Model 3 were 63.56 MPa and 62.65 MPa, respectively. The stress values on the multi-unit abutments after the vertical loading were not significantly different between the models. As a result, the elasticity modulus of the bar attachment material did not affect the stress borne by the multi-unit abutment.

### 3.3. Horizontal Loading

**Figure 5:**
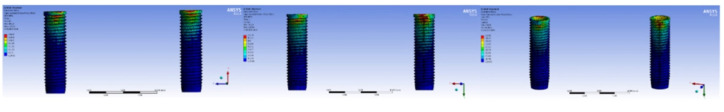
The Von Mises stress distribution on Model 1, Model 2 and Model 3 implants after horizontal loading.

According to the results, the highest Von Mises stress levels were indicated in Model 1 (419.42 MPa). The Von Mises stress distribution on the implants in Model 1, Model 2 and Model 3, respectively, after horizontal loading is shown in Figure 5. Model 3 (316.68 MPa) and Model 2 (312.24 MPa) follow Model 1 stress values. The necks of the implants are the densely stressed parts. The amount of Von Mises stress on the implants was in the range of Ti yield strength. This was why no deformation occurred.

**Figure 6:**
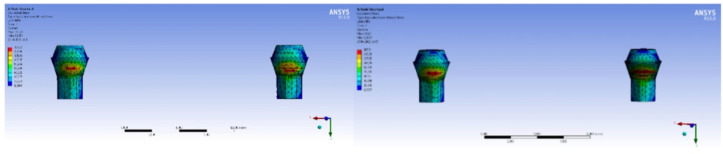
The Von Mises stress distribution on Model 2 and Model 3 multi-unit abutment after horizontal loading.

Since the load was applied from only one point on the bar attachment, specific areas bore more stress. These specific areas were ignored, and the conclusion was reached that loading did not have any significant effect on the bar attachment. The Von Mises stresses on the multi-unit abutments in Model 2 and Model 3 were 171.72 MPa and 365.5 MPa, respectively. Figure 6 exhibits the Von Mises stress distribution on the multi-unit abutments in Model 2 and Model 3, respectively, after horizontal loading. The stress on the multi-unit abutments was mostly seen around the neck part in both models. The type of bar attachment material significantly affected the stress on the multi-unit abutments. The elasticity modulus of Co-Cr alloy was high, and on account of this, the stress increased in Model 3.

### 3.4. Oblique Loading

**Figure 7:**
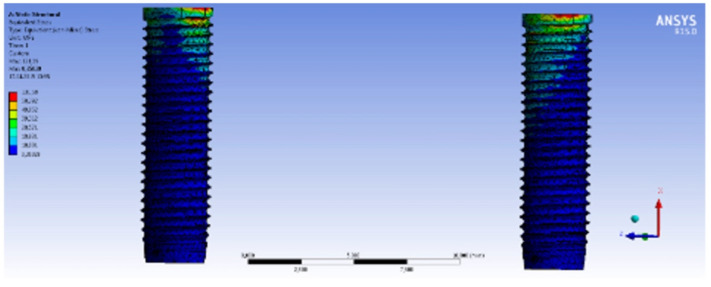
Model 1 implants and the Von Mises stress distribution after oblique loading.

**Figure 8:**
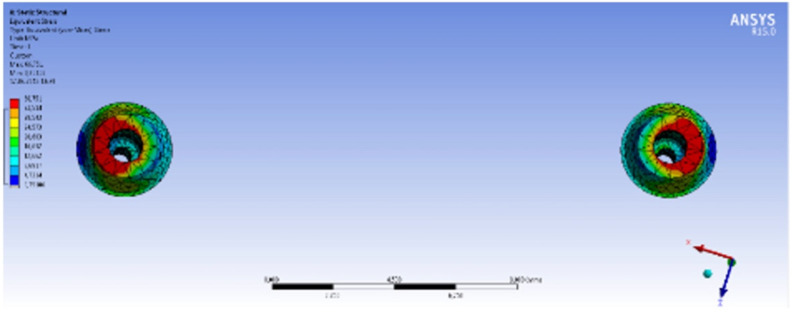
Model 2 multi-unit abutment and the Von Mises stress distribution from occlusal view after oblique loading.

The highest Von Mises stress on the implants at oblique loading was observed in Model 1 (131.59 MPa). Figure 7 exhibits the implants and the Von Mises stress distribution after oblique loading in Model 1. The lowest Von Mises stress levels were indicated in Model 3 (87.66 MPa). According to these results, multi-unit abutment provides stress absorbance, showing lower stress on the implants. The stress was observed on the neck of the implants and the area just across the place where the load was applied. The stress on the implants after the oblique loading on each model was in the range of the Ti yield strength values, and that is the reason why no deformation occurred. The Von Mises stress on the multi-unit abutment was 66.75 MPa in Model 2, and 55.4 MPa in Model 3. Figure 8 shows the multi-unit abutment and the Von Mises stress distribution from an occlusal view after oblique loading.

In all loading conditions, the Von Mises stress values of the trabecular bone were lower than the values of the cortical bone, as the elasticity modulus of the trabecular bone is lower than the elasticity modulus of the cortical bone.

### 3.5. The Minimum and Maximum Principal Stress on the Cortical Bone

The Minimum and Maximum Principal Stress values on the cortical bone tissue for all models in vertical, horizontal and oblique loading of 100 N are indicated in Table 4. In all of the loading conditions, Model 1 showed the lowest minimum principal stress value. Considering the absolute value of the minimum principal stress values, Model 1 showed the highest compressive stress occurrence in the cortical bone. Model 2 showed the highest maximum principal stress values in vertical loading. However, in horizontal and oblique loading, the highest maximum principal stress values belonged to Model 1. Considering all three models, the highest maximum principal stress values and the lowest minimum stress values were observed at horizontal loading condition.

## 4. Discussion

Implant-retained overdenture treatment for total edentulous cases is becoming popular, as technological developments in dentistry become widespread. The long-term success of the implants depends on the load transmission to the supporting tissues. The stress transmission from the implants to the neighbouring tissues may alter, depending on several factors: the amount and direction of the applied load; the diameter, length, number and surface characteristics of the inserted implants; the bone-implant interface; the type of the prosthesis and the bone quality. Thus, the biomechanical properties of the bone-implant connection must be examined carefully [51,52,53].

In their study, Saito, et al., analysed the retentive forces of various bar materials, and pointed out that Ti and Co-Cr exhibited more stiffness and higher wear resistance than Au-Pd alloy [54]. With its high stiffness, Co-Cr alloys demonstrated more stress compared to other metal alloys in most of the studies [16]. As Ti Grade 5 and Co-Cr frameworks are often preferred in clinical practice, there is a trend towards also using these materials as metal bar frameworks in in vitro studies. Unfortunately, casting of Ti alloys is challenging because of their affinity to gases, their high melting point and their being over-reactive to the investment materials. Consequently, milling of Ti alloys is preferable [55]. However, because CAD/CAM technology and the milling method have the disadvantage of high costs, the conventional casting method is still widely preferred in clinical practice. Co-Cr alloys are favourable in bar framework fabrication, having the advantages of low cost, castability and material hardness [56]. Therefore, milling of Ti alloy and conventional casting of Co-Cr was preferred in this study.

Castolo, et al., conducted research comparing implant-supported overdentures with different scenarios, including different bar materials such as Ti Grade 4, Ti Grade 5, zirconium oxide and Co-Cr. As with our study, the researchers stated that material hardness had did not significantly affect on stress occurrence on the implants, and that the stress borne by the bone carried no physiological risk [57]. However, in their study, Caetano, et al., stated that material stiffness had a definite effect on increasing the stress values [58].

Tabata, et al., compared the stress distribution in a 2-implant mandibular overdenture prosthesis with bar-clips and with O-ring attachments, respectively, using the 2D finite element analysis, and determined that the results were higher with the O-ring attachment than with the bar-clips. The study concluded that the bar-clips retentive system was successful in stress distribution to the bone tissue around the implant [59].

The application of static loads rather than dynamic loads, and accepting the vital tissues as isotropic, are the two main limitations in 3D-FEA studies [34]. Furthermore,, bone is a complex vital structure, which varies in different individuals [60]. That is why this method should not be considered on its own. Although there are limitations to the 3D-FEA method, it is more advantageous than in vivo research methods, since the researcher has the opportunity to change the model geometry, loading type and boundary conditions manually on the computer. Moreover, the repeatability of this method is a great advantage compared to in vivo techniques [61].

Menicucci, et al., compared the stress distribution on the bone around the implant with the action and reaction force seen in the distal part of the total edentulous jaw in 2-implant retained overdentures with bar-clips and ball attachment retentive systems, using 3D finite element analysis. The reaction forces on the distal edentulous mucosa, and the stress on the peri-implant bone, were compared in overdentures retained either by two ball attachments or by two clips on a bar connecting two implants. In the finite element model, a 35 N load on the first mandibular molar induced a greater reaction force on the distal edentulous mucosa of the non-working side when the overdenture was anchored by ball attachments, than with the clips/bar attachment. Stress on the peri-implant bone was greater with the clips/bar attachment than with the ball attachment [41]. According to these results, there is no common decision on whether ball or bar system is better on load transmission.

In this study, a single unit bar design was preferred for use, as having a better load transmission in the whole system. The bar attachment prevents the vertical and oblique forces transmission towards the direction of displacement. Retention and stability were better than in the individual attachments, since the system behaved like a whole unit [41,59]. The biomechanical factors that affect the bar-clip attachment system are: the number of implants, the properties of the fabrication material and the length of the bar attachment [49]. The shape of the bar system also changes the stress distribution. Stress-breaking attachments like Hader Bar are preferable to the rigid, oval-shaped Dolder Bar, regarding better stress distribution in peri-implant tissues [40,62]. The Hader Bar provides hinge movement around only one rotation axis. By means of splinting the implants, the individual mobility of the implants is prevented [59]. Due to its advantages, the Hader Bar attachment design was chosen to be used in this research, and a comparison of the stress distribution was made over various fabrication methods with different materials.

Traditional casting, laser sintering of prefabricated components, CAD/CAM and CNC milling are the manufacturing methods of bar-retained overdenture restorations [63,64,65]. The fabrication of bar attachments with traditional casting method and lost-wax technique may cause an implant-bar attachment misfit due to volumetric changes, and linear contraction and expansion occurring in the impression material, dental stone, wax, investment and cast metal alloy. The potential of distortion is eliminated in the CNC Milling machine during milling of a monoblock Ti block [66,67].

The CNC Milling method provides the fabrication of the bar attachment, eliminating the problems originating from the patient, the surgical procedure and implant companies without a multi-unit abutment. The direct CNC Milling technique is used when either the implants are malpositioned, and even the multi-unit abutment cannot tolerate the angular difference, or when the implants are inserted too deep and the implant company cannot provide the proper gingival height multi-unit element. Moreover, the CNC Milling technique provides a custom bar attachment fabrication by transferring models to the computer and using specific software in cases where neither the dentist nor the patient has a knowledge of the implant company. In some comparative studies of CNC Milling technique and the traditional casting method, it has been determined that CNC Milling exhibits a better adaptation between the framework and the implants [68,69].

The misfit between the system tools related to the bar attachment manufacturing causes bacterial invasion, peri-implantitis and crestal bone resorption [69,70]. Caetano, et al., studied vertical misfit as a variable in their research by creating a three-dimensional jaw model and an over-denture retained by two implants splinted with a bar. They concluded that the higher vertical misfit affected the stress values negatively, especially with a stiff framework [58]. In this study, a perfect-fit bar attachment connection was considered.

In the literature, there are no studies including a comparison of the mechanical strength of bar retentive systems fabricated with traditional casting, and the CNC Milling method. For this reason, in this study, the stress distribution on the screw and the bar system was examined in addition to implants and the peri-implant bone.

In their study, Spazzin, et al., researched the horizontal misfit effect and the bar material effect. Four models were created with different levels of horizontal misfit (10, 50, 100, 200 μm) between bar and implant, using Au as the bar material. For the bar material effect, four models were created using different bar materials (Au, Ti, Ag–Pd, Co–Cr) with a 50 μm horizontal misfit between the bar and the implant. The misfit amplification presented a great increase in the stress levels in the inferior region of the bar, in the screw-retaining neck, in the cervical and medium third of the implant, and in the cortical bone tissue surrounding the implant. The higher stiffness of the bar presented a considerable increase in the stress levels in the cortical bone tissue and bar framework, while the retaining screw and implant presented few changes in stress values [44,71]. Unlike this study, Natali et al., pointed out that the resilience of the bar material provided a decrease in the stress transmitted to the bone surrounding the implant [72].

In their research, Meijer et al., studied 3D-FEA [73] and, according to them, under realistic loading and boundary conditions the stress distribution results obtained from the mandibular interforaminal region models can represent the entire mandible model results. Therefore, 3D mandibular interforaminal region models were preferred in this study. Furthermore, in another study, the same researchers advised that increasing the number of nodes and elements eventuates more realistic results [74].

The average of the masticatory forces in removable denture wearers are in the range of 100 N and 140 N [49,75] in the literature. Sharma, et al., and Helmy and Kothayer, stated that the maximum masticatory bite force for complete denture wearers is 60–80 N. These values increase to 150–170 N for patients with implant-supported overdenture [76,77]. In a study by Hu, et al., the moderate level of average masticatory occlusal force for the implant overdentures was stated to be 100 N [78]. Based on these previously published data, 100 N of static loads were applied to simulate the average moderate masticatory forces.

Miscellaneous studies with FEA have included posterior loading with different criteria, unlike this study. Nevertheless, the places and number of the implants were similar to this research, which was two implants inserted in the interforaminal region [34,35,36,38]. Four implants with ball attachments and a bar attachment on the maxilla were studied separately with FEA by Geramy and Habibzadeh [37]. Splinting of the implants was advised, based on their results. In another study, two implants in the mandibular interforaminal region were splinted with a bar, 60 N of load was applied at the centre of the bar, and 100 N was applied at the end of the bar bilaterally. The Von Mises stress values were higher in the second incisor placement rather than in the canine. In this study, the most common basic treatment for the patients was preferred [79].

## 5. Conclusions

From this study, the following can be concluded:

- The highest Von Mises stress on the implants and the bone tissue was observed in all models after 100 N of horizontal loading. The horizontal loading stress values were higher than the vertical and oblique loading stress values.
- In all loading conditions, the highest Von Mises stress on the implants and the cortical bone was observed in Model 1, which was constructed with no multi-unit abutment. Using multi-unit abutments in bar-retentive overdenture systems provides stress absorption, and this results in low tensile stress values in the implants and the bone.
- The elasticity modulus of the bar material did not have a significant effect on stress occurrence in implants and bone.
- After the horizontal, vertical and oblique loading, the stress on the implants, and on the cortical and trabecular bone in the Co-Cr bar system with multi-unit abutments, was nearer to, or lower than, the values of the Ti Grade 5 (Ti-6Al-4V) bar system. This is connected to the use of the manufacturer’s original implant multi-unit abutments.
- After the implementation of static load in three different directions, the maximum principal stress in the cortical bone was lower than the tensile and the compressive strength values of the bone. The stress occurrence on the trabecular bone was lower than the stress occurrence on the cortical bone.

## 6. Clinical Implication

In this study, stress distribution related to various fabrication techniques and materials in Hader Bar-retained mandibular overdentures was evaluated. The use of multi-unit abutments in overdenture prosthesis has an advantage for stress distribution. The diversity in the bar material does not affect stress occurrence in the implants and bone. While planning a 2-implant-retained mandibular overdenture prosthesis, with standard gingival height conditions, bar fabrication with an original multi-unit abutment from the manufacturer can be preferred. Attention should be paid in case of any load in a horizontal direction. However, for more precise results, more studies and clinical practice is needed.

## Figures and Tables

**Table 1:** The elasticity modulus and the Poisson ratio of each material used in this study.

Material	Poisson Ratio (V)	Elasticity Modulus (MPa)
Cortical Bone [41,42,43,44]	0.3	13,700
Trabecular Bone [41,42,43,44]	0.3	1370
Mucosa [41,42]	0.37	1
İmplant [33,43]	0.33	110,000
Screw [43,44]	0.28	110,000
Bar (Ti Grade 5/Ti-6Al-4V) [45,46]	0.35	103,400
Bar (Co-Cr alloy) [44]	0.33	218,000
Multi-unit abutment (Ti-6Al-7Nb) [46,47]	0.28	110,000

**Table 2:** The tensile strength and the yield strength of the materials used in this study.

	Yield Strength (MPa)	Tensile Strength (MPa)
Ti [50]	680	760
Ti-6Al-4V (Ti Grade 5) [50]	760	930
Cortical Bone [50]	--	88–164
Trabecular Bone [50]	--	23

**Table 3:** The Von Mises stress values on implants, cortical bone and trabecular bone after the application of 100 N static load.

100 N				
Model	Loading Direction	Stress Values (MPa)		
		Implant	Cortical Bone	Trabecular Bone
Model 1	Horizontal	419.42	119.60	2.84
	Vertical	148.24	19.92	1.62
	Oblique	131.59	32.21	1.68
Model 2	Horizontal	312.24	72.10	3.27
	Vertical	87.49	20.09	1.75
	Oblique	105.10	20.44	1.57
Model 3	Horizontal	316.68	72.14	3.26
	Vertical	86.86	19.95	1.75
	Oblique	87.66	19.10	1.45

MPa = Megapascal; N = Newton.

**Table 4:** The Maximum and Minimum Principal Stress values on the bone tissue after 100 N of horizontal, vertical and oblique loading.

100 N			
Model	Loading Direction	Principal Stress Values (MPa)	
		Maximum Principal Stress	Minimum Principal Stress
Model 1	Horizontal	141.69	−114.75
	Vertical	9.77	−23.73
	Oblique	31.34	−36.33
Model 2	Horizontal	61.50	−84.47
	Vertical	12.99	−20.74
	Oblique	7.30	−20.14
Model 3	Horizontal	61.97	−83.95
	Vertical	12.93	−20.60
	Oblique	6.98	−19.00

MPa = Megapascal.
